# Short-term predictive potential of quantitative assessment of spinal cord impairment in patients undergoing French-door Laminoplasty for degenerative cervical myelopathy: preliminary results of an exploratory study exploiting intraoperative ultrasound data

**DOI:** 10.1186/s12891-020-03319-w

**Published:** 2020-05-30

**Authors:** Guoliang Chen, Jiachun Li, Fuxin Wei, Qiao Ji, Wenyuan Sui, Bailing Chen, Xuenong Zou, Zuofeng Xu, Xizhe Liu, Shaoyu Liu

**Affiliations:** 1 Department of Orthopedic Surgery, The Seventh Affiliated Hospital, Sun Yat-sen University, Shenzhen, P.R. China; 2 Guangdong Provincial Key Laboratory of Orthopaedics and Traumatology /Orthopaedic Research Institute, Department of Spine Surgery, The First Affiliated Hospital, Sun Yat-sen University, No.58 Zhongshan 2nd Road, Guangzhou, 510080 P.R. China; 3 Department of Ultrasound, The Seventh Affiliated Hospital, Sun Yat-sen University, No.628 Zhenyuan Road, Shenzhen, 518107 P.R. China

**Keywords:** Degenerative cervical myelopathy, French-door laminoplasty, Gray value, Intraoperative ultrasound, Magnetic resonance imaging

## Abstract

**Background:**

To study the correlation of neurological function in degenerative cervical myelopathy (DCM) patients with quantitative assessment of spinal cord compression and impairment by intraoperative ultrasound imaging (IOUSI).

**Methods:**

Twenty-three patients who underwent French-Door laminoplasty for multilevel DCM were followed for 6 months. Modified Japanese Orthopaedic Association (mJOA) score and cervical MRI were assessed before surgery and at postoperative 6 months. IOUS, used to guide decompression, were recorded. The anteroposterior diameter (APD) and the gray values of the IOUSI hyperechogenicity of the midsagittal IOUSI at the narrowest level and at the lesion-free level, and the APD and traverse diameter at the traverse maximum compression level of IOUSI were measured. Maximum spinal cord compression (MSCC), compression rate (CR), and IOUSI gray value ratio (R_gray_) were calculated. The appearance of preoperative T2W MRI increased signal intensity (ISI), and the signal change rate (SCR) on postoperative T2W MRI of 9 patients were also measured and calculated, and compared with that of IOUSI hyperechogenicity.

**Results:**

Average mJOA score increased significantly from 11.57 ± 2.67 before surgery to 15.39 ± 1.50 at 6 months after surgery, with an average recovery rate (RR) of 71.11 ± 22.81%. The difference between the appearance of preoperative T2W MRI ISI and IOUSI hyperechogenicity was not significant. Spearman correlation analysis found that the IOUSI R_gray_ were negatively correlated with the RR of mJOA score with a coefficient of − 0.77, and the IOUSI R_gray_ was not correlated with the postoperative MRI SCR.

**Conclusions:**

In DCM patients, the gray values of IOUSI can be measured accurately. The IOUSI R_gray_ correlated with postoperative neurological recovery significantly.

## Background

Degenerative cervical myelopathy (DCM) which leads to spinal cord compression is the most common cause of spinal cord dysfunction in adults worldwide [1–3]. Magnetic resonance imaging (MRI), which can indicate the degree of spinal stenosis and reveal the pathological status of the spinal cord in detail, is an important and effective method for the diagnosis and prediction of DCM [4, 5]. The MRI indices widely used to evaluate the compression and impairment of the spinal cord in DCM include the maximum spinal cord compression (MSCC), compression ratio (CR), signal intensity and signal change rate (SCR) on T2W MRI, many studies revealed that above indices made different contributions to the diagnosis and prediction of DCM, but still some studies considered the effect of these features was unclear [6–9]. Recently, due to its non-invasive nature and real-time imaging, intraoperative ultrasound (IOUS) has become the preferred method to use during cervical laminoplasty to guide decompression and to evaluate the relationship between the degree of decompression and postoperative neurological function [10–18]. Interestingly, IOUS imaging (IOUSI) also showed different MSCC, CR and echogenicity (brightness) of spinal cord by degree of compression. However, the correlations between the above ultrasonic manifestations of the spinal cord and neurological function has not been reported. IOUS detected the real status of the spinal cord after decompression, resulting in a measurement more similar to the postoperative situation. We speculate that those intraoperative ultrasonic manifestations can be also used to predict the postoperative neurological function in DCM. In order to verify our hypothesis, we conducted a clinical study with short-term follow-up to analyze the correlation between short-term outcome of French-door laminoplasty (FDL) and intraoperative ultrasonic manifestations.

## Methods

### Study population

This study followed the principles outlined in the Declaration of Helsinki and was approved by The Seventh Affiliated Hospital of Sun Yat-sen University Ethics Committee. Written informed consent was obtained from all participants in the study. A total of 26 patients with multilevel DCM (≥3) were consecutively and prospectively enrolled between October 2018 and May 2019. Patients with a history of thoracic or lumbar disorders, trauma, infection, tumor, rheumatoid arthritis or previous cervical surgery were excluded. In total, 23 patients (17 males and 6 females) followed for at least 6 months were included in this study because many studies show that the recovery of spinal cord function is stable within 6 months after cervical laminoplasty [19–21]. All the 23 patients obtained preoperative MRI and satisfactory IOUSI. Additionally, for 9 patients finished the postoperative MRI, we also preliminarily analyzed the correlation between the manifestations of postoperative MRI and IOUSI.

### Surgical technique and IOUS

**Fig. 1:**
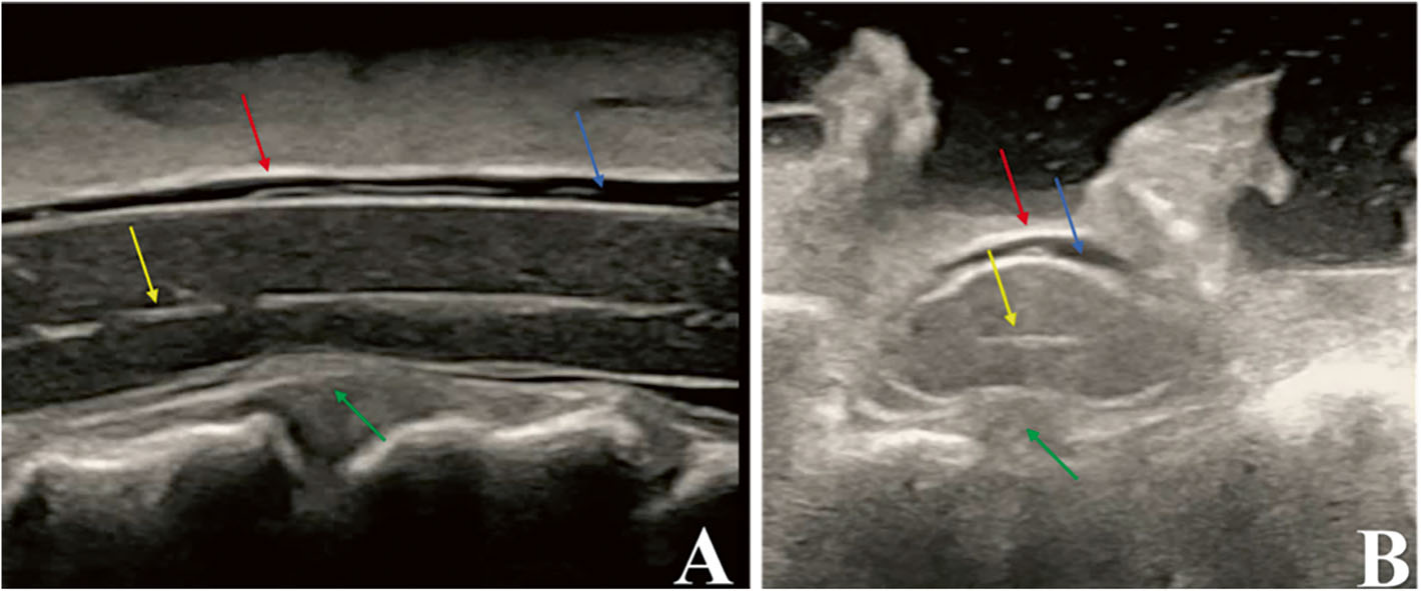
The anatomy of the spinal cord observed by intraoperative ultrasound. **a**) Midsagittal image, (**b**) Axial image. Red arrows indicate the spinal dura mater, yellow arrows indicate the central canal, blue arrows indicate the subarachnoid space and green arrows indicate the cervical disc

All patients received FDL from the same chief spine surgeon, performed according to Kurokawa’s method [22] with a few modifications. After detachment of the bilateral paravertebral muscles from the spinous processes, the centers of spinous processes were cut using a fretsaw. Bilateral gutters were created as hinges at the border of the laminae and facets. After the halves of the laminae were elevated using an expander, normal saline was infused into the operation area and formed an acoustic window, then a 14.0 MHz linear array transducer of IOUS (M7Expert, Mindray Medical International Limited, Shenzhen, China) was used to observe the spinal cord and record the images (Fig. 1). If residual compression was observed, further decompression under the guidance of IOUS was done. After the decompression was finished, normal saline was used to remove debris, then appropriately-sized hydroxyapatite spacers were tied in place to bridge the bilateral edges of the laminae and fixed with wires. Finally, a drainage tube was placed and the wound was closed in layers.

### Postoperative considerations

All patients were allowed to walk while wearing a fitted collar from postoperative day 1. The collar could be removed at the patient’s discretion. Cervical exercises were performed from postoperative day 1. All patients were followed up regularly at 1 month, 3 months and 6 months after surgery.

### Clinical assessments

The surgical procedures were evaluated in terms of the operative time, blood loss and perioperative complications. Neurological function was evaluated using the modified Japanese Orthopaedic Association (mJOA) score before surgery and at each follow-up. The recovery rate (RR) according to the mJOA score was calculated at postoperative 6 months using the following formula:

*RR of mJOA score = (Postoperative mJOA score – Preoperative mJOA score)/ (17 – Preoperative mJOA score) × 100%.*

### Radiological assessments

The anteroposterior diameter (APD) and traverse diameter (TD) were evaluated using Adobe Photoshop (Adobe Systems, San Jose, CA, USA). The gray value of hyperechogenicity can be quantified by ImageJ software (National Institutes of Health, Bethesda, MD, USA). The appearance and signal intensity of increased signal intensity (ISI) on T2W MRI (generated from 1.5 T MRI scanner, GE Healthcare, Chicago, IL, USA) were evaluated and measured using the MRI workstation (DJ HealthUnion Systems Corporation, Shanghai, China). All patients’ images were assessed by the same two researchers independently and repeated three times, the mean was used for statistic analyzing.

On IOUSI, the midsagittal APD (the midsagittal slice was determined by the visualization of the central canal of spinal cord) at the narrowest level (APD_min_) and the compression-free level (APD_normal_) were measured and then calculated the MSCC (*MSCC = APD*_*min*_*/APD*_*normal*_). The APD_traverse_ and the TD of the narrowest traverse slices were measured and then calculated the CR (*CR = APD*_*traverse*_*/TD*).

In order to avoid the deviations caused by variations between machines and operators, we defined the gray value ratio, R_gray_, by calculating the ratio of the gray value in the midsagittal IOUSI at the narrowest level to that at the lesion-free level: *R*_*gray*_ *= Gray*_*narrowest*_*/Gray*_*normal*_*.*

**Fig. 2:**
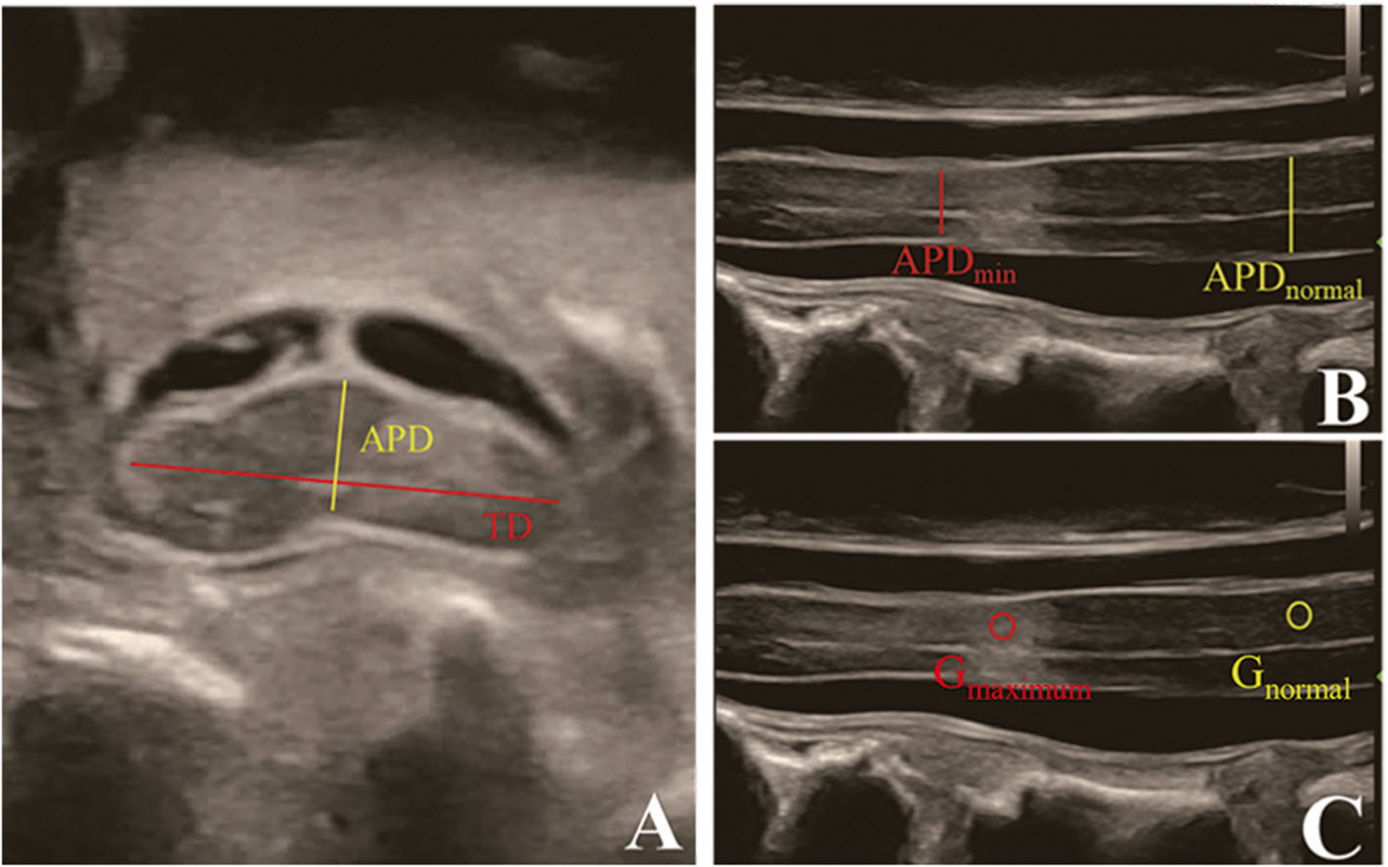
**a** Traverse images at the maximum level of patient with degenerative cervical myelopathy (DCM), depicting the measurements required to calculate the compression ratio (CR). Measurements for CR of intraoperative ultrasound imaging (IOUSI), including the anteroposterior diameter (APD) and the traverse diameter (TD) of the spinal cord. **b**) Midsagittal IOUSI of patient with DCM, depicting the measurements required to calculate maximum spinal cord compression (MSCC). Measurements for MSCC of intraoperative ultrasound imaging (IOUSI), including the width of the spinal cord at the narrowest site (APD_min_) and the width of the spinal cord at the normal site (APD_normal_). **c** Midsagittal IOUSI of patient with DCM, depicting the measurements required to calculate the gray value ratio (R_gray_). A midsagittal intraoperative ultrasound imaging (IOUSI) of a patient, the measurements required to calculate the gray value ratio (R_gray_), including a 0.1 cm^2^ measurement of the gray value at the site of the maximum compression level of the spinal cord (G_maximum_) and 0.1 cm^2^ gray value measurements at the lesion-free site (G_normal_)

The IOUSI R_gary_ and was measured according to the methods described in previous studies about the measurement of signal change rate on T2W MRI with few modifications [7, 21]. In brief, for patients with macroscopic hyperechogenicity on IOUSI, a circle of 0.1 cm^2^ was drawn with the maximum brightness point as the center, and a circle of 0.1 cm^2^ was drawn on the lesion-free level, then the gray values of both circles were measured by ImageJ respectively, and calculated the R_gray_. For patients without different visible brightness within the spinal cord, a circle of 0.1 cm^2^ was drawn within the maximum compression level, and another circle of 0.1 cm^2^ was drawn on the lesion-free level, then measured the gray values and calculated the R_gray_. All circles avoided involving the central canal of spinal cord. (Fig. 2).

For the 9 patients with postoperative MRI, we also measured the signal intensity of ISI and calculated the SCR of T2W MRI and then analyzed the correlation between the IOUSI R_gray_ and SCR of T2W. In brief, the measurement of the signal intensity of ISI was in line with that of IOUSI, but the reference standard was the signal intensity of cerebrospinal fluid (CSF) measured on the cisterna magna. The signal intensity values were generated from the MRI workstation, and calculated the SCR (*SCR = Signal*_*narrowest*_*/ Signal*_*CSF*_).

### Statistical analysis

Data were analyzed using SPSS statistical software version 24.0 (ICM Corp., Armonk, NY, USA). All values were expressed as mean ± standard deviation (SD). Paired t test was used to compare the differences between pre- and post-operative mJOA scores, Chi-square test was used to compare the displaying rate of preoperative T2W MRI ISI and the IOUSI hyperechogenicity, Spearman correlation analysis was used to analyze the correlation between the IOUSI MSCC, CR, R_gray_ and the preoperative mJOA; between the IOUSI MSCC, CR, R_gray_ and the RR of the mJOA score; and between the postoperative T2W MRI SCR and the IOUS R_gray_. *P* values less than 0.05 were considered to be statistically significant.

## Results

The mean age at surgery was 62.09 ± 11.80 years, the mean operative time was 196.18 ± 18.66 mins and the mean blood loss was 190.91 ± 181.41 ml. The average mJOA score increased significantly from 11.57 ± 2.67 before the operation to 15.39 ± 1.50 at 6 months after surgery (*P* < 0.001), and the average RR of the mJOA score was 71.11 ± 22.81%. No complications were reported at 6 months after surgery. (Table 1).

**Table 1:** Clinical and radiological data

Indicator	Result
number of cases	23
male	17
female	6
age at surgery (years)	62.09 ± 11.80
blood loss (ml)	190.91 ± 181.41
operative time (min)	196.18 ± 18.66
preoperative mJOA score	11.57 ± 2.67
mJOA score at postoperative 6 months	15.39 ± 1.50*
the RR of mJOA score (%)	71.11 ± 22.81
R_gray_ of IOUSI	2.12 ± 0.42
correlation coefficient of R_gray_ and the RR of mJOA score	−0.77 (*P* = 0.004)

*mJOA score* modified Japanese Orthopaedic Association score, *RR* recovery rate, *R*_*gray*_ the gray value ratio, *IOUSI* intraoperative ultrasound imaging, *SCR* signal change rate

*: compared with the preoperative mJOA score, *P <* 0.001

**Fig. 3:**
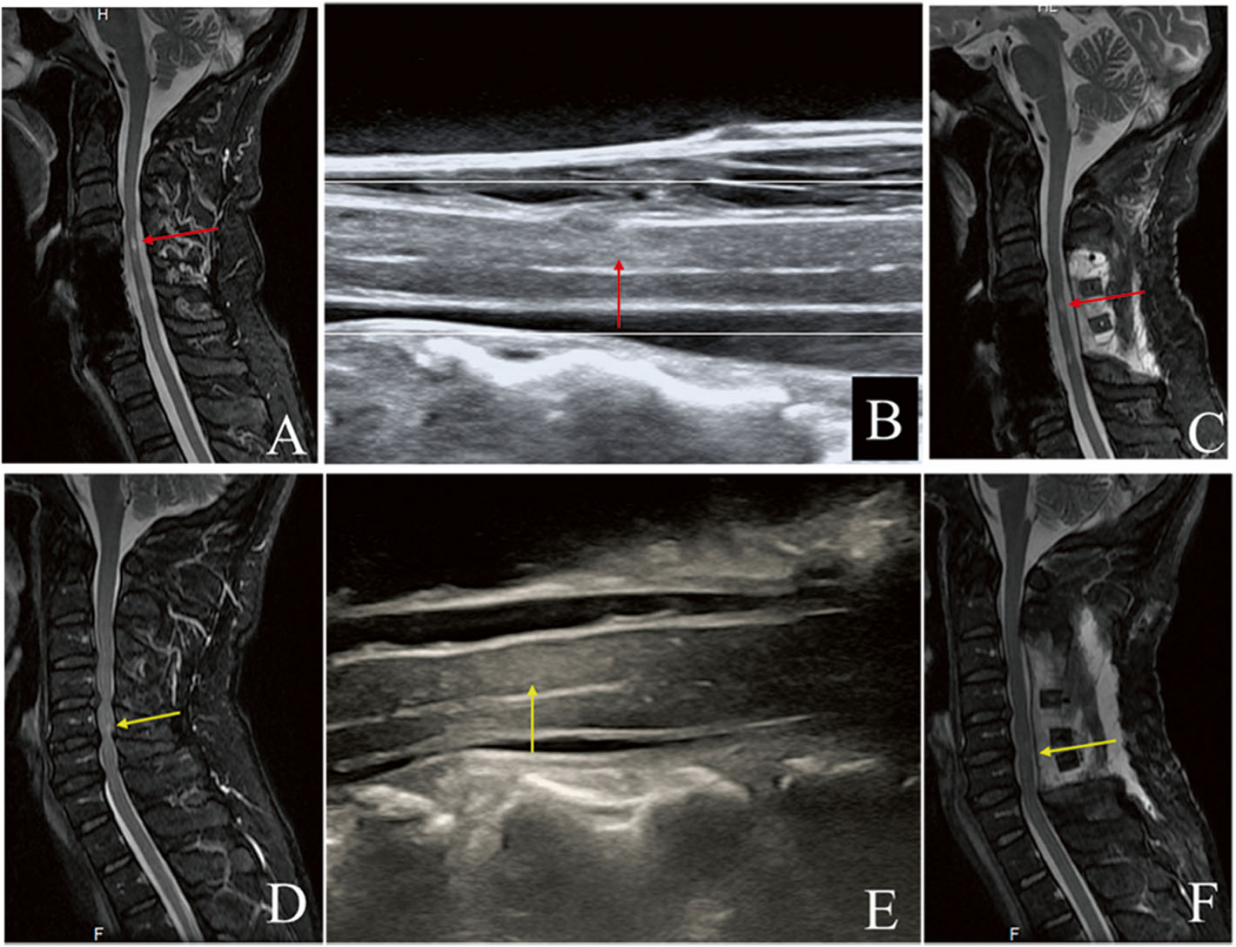
The preoperative T2W MRI, IOUSI and postoperative T2W MRI of midsagittal spinal cord of two cases (case 1: A-C, case 2: D-F). The positions of IOUSI hyperechogenicity were in line with that of preoperative T2W MRI increased signal intensity (ISI). But for the patient (case 1) with relatively low IOUSI hyperechogenicity, the postoperative ISI on T2W MRI was still existed while the patient (case 2) with high IOUSI hyperechogenicity, the postoperative ISI on T2W MRI was disappeared

The preoperative T2W MRI ISI and IOUSI hyperechogenicity were detected in 20 patients simultaneously. For those patients, the positions of IOUSI hyperechogenicity were in line with that of T2W MRI ISI (Fig. 3). One patient showed hyperechogenicity on IOUSI but no ISI on T2W MRI while two patients showed neither T2W MRI ISI and IOUSI hyperechogenicity. Totally, hyperechogenicity was detected in 21 patients while T2W MRI ISI was detected in 20 patients, with a displaying rate of 91.30% (21/23) and 87.96% (20/23) respectively. The difference of the displaying rates of those two methods was not statistically significant. (Table 2).

**Table 2:** The appearance of IOUSI hyperechogenicity and T2W MRI ISI

	with T2W MRI ISI	without T2W MRI ISI	total
with IOUSI hyperechogenicity	20	1	21
without IOUSI hyperechogenicity	0	2	2
total	20	3	23

*IOUSI* intraoperative ultrasound imaging, *T2W MRI ISI* T2-weighted magnetic resonance imaging increased signal intensity

The average R_gray_ of IOUSI of 23 patients were 2.12 ± 0.42. Spearman correlation analysis showed that the IOUSI R_gray_ was negatively correlated with the RR of mJOA score, with a correlation coefficient of − 0.77 (*P* = 0.004). Values of the indexes of compression, such as the MSCC or the CR, showed no correlation with preoperative mJOA score or the RR of mJOA score. Neither was there a correlation of the IOUSI R_gray_ with the preoperative mJOA score.

For the 9 patients with postoperative MRI, the average IOUSI R_gray_ and SCR of T2W MRI was 1.90 ± 0.35 and 0.76 ± 0.15 respectively. Spearman correlation analysis showed that there was no correlation between IOUSI R_gray_ and SCR of T2W MRI, with a correlation coefficient of 0.20 (*P* = 0.606). (Fig. 3).

## Discussion

In this study, quantitative measurements of IOUSI were used to evaluate the compression and impairment of the spinal cord in DCM. We found no correlation between the indexes of spinal cord compression (MSCC and CR) and the mJOA score, but a strong correlation between the index of spinal cord impairment (R_gray_) and the mJOA score.

Although many studies have examined the correlation between compression and neurological function, the results remain controversial [23–25]. In our clinical experience, many patients show severe spinal cord compression on preoperative MRI, but have no clinical symptoms, or patients with the same degree of compression show different clinical symptoms. The results of this study also confirmed this experience, that there is no correlation between the IOUSI indexes of spinal cord compression and the mJOA score. These findings are similar to those of many previous studies. Hiroaki et al. [23] conducted cervical MRI scans on 1211 volunteers without cervical myelopathy, and found that 64 cases had spinal cord compression of different degrees. The most severe case had a 77.6% decrease in the cross-sectional area of the spinal cord at the C5/6 level, leading the authors to believe that a significant degree of spinal cord compression can be tolerated without any symptoms. Alina et al. [25] found, through systematic review, that indicators such as CR and MSCC are commonly used to evaluate spinal cord compression, but the relationship between these indicators and spinal cord function is still controversial. Compression may be an initiator of spinal cord impairment, but the real cause of impairment may be the pathological alterations such as spinal parenchymal edema, ischemia or cysts secondary to compression [26, 27].

T2W MRI ISI of the spinal cord is often thought to be caused by parenchymal edema, ischemic and cystic degeneration of the spinal cord [26]. Interestingly, we found that many cases showed intramedullary hyperechogenicity change at the lesions of the spinal cord on IOUSI. The position of hyperechogenicity areas on IOUSI were in line with the ISI on T2W MRI, and the displaying rates of IOUSI hyperechogenicity and T2W MRI ISI showed no significant difference. Based on these findings, we hold the opinion that like T2W MRI ISI, the intramedullary hyperechogenicity on IOUSI could also reflect the impairment of spinal cord. But our results showed no correlation between the IOUS R_gray_ and the SCR of the postoperative T2W MRI. To our knowledge, the imaging principles of IOUS and MRI are quite different, and the timing of IOUS and postoperative MRI and the compressive status of the spinal cord are also quite different. These differences may explain why the IOUSI R_gray_ was not correlated to the SCR of T2W MRI.

IOUS has been used in spine surgery since the 1980s, [28] and is widely used to evaluate and guide the decompression of the cervical spinal cord during surgery. However, to our best knowledge, most current studies have focused on the correlation between the morphological changes after decompression in the spinal cord, the degree of decompression and the postoperative neurological function [12–18]. The correlation between neurological function and the gray value of IOUSI, which may reflect the impairment of the spinal cord, has not been reported. As shown in Fig. 3, the compressive area manifests brighter than the non-compressive areas on IOUSI, with the varying brightness indicating varying gray values. The gray values of the compressive area were higher than those of the non-compressive area, a finding similar to that of a previous animal study [29]. In addition to using IOUS to guide decompression, we also measured the gray value of IOUSI at the most compressed and non-compressed segments of the spinal cord to calculate the R_gray_, and analyzed the correlation between IOUSI R_gray_ and the RR of mJOA score. The result revealed that the IOUSI R_gray_ correlated with the surgical outcome significantly.

Ultrasonography is a common diagnostic method which uses sound-wave pulses transmitted into tissue to analyze the temporal and sound properties of the reflected wave. Echoes occur at tissue interfaces, with the reflected energy related to the differences in density of the adjacent tissues (acoustic impedance) as well as the angle of the ultrasound beam (angle of incidence) into the interface [30]. A fraction of the transmitted sound-wave energy is reflected at each change in the acoustic impedance within the tissue. Greater differences in the density of adjacent tissues result in larger echoes. As a result, a picture is formed from the different gray values according to the echoes received by probe. The pathophysiology of spinal cord lesion hyperechogenicity, like the ISI of the lesion on T2W MRI, is incompletely understood. According to existing views, DCM is caused by static and dynamic mechanical forces acting on the spinal cord. Chronic spinal cord static compression causes segmental ischemia, edema, neuron apoptosis, spinal cord atrophy and cystic necrosis. In the flexion and extension states of the cervical spine, dynamic repetitive injuries further stretch the axons [31, 32]. Based on previous studies, we assumed that the hyperechogenicity in the IOUSI is caused by spinal cord edema, the proliferation of fibroblasts and capillary endothelial cells, fibrin deposition, the damming of nerve fiber transport, the invasion of mast cells and macrophages, and finally, fibrosis [33, 34]. These pathological changes make the density of the spinal cord uneven and, according to the imaging principles of ultrasound, the damaged spinal cord then shows different brightness and gray values on the IOUSI. Our results revealed a strong negative correlation between the IOUSI R_gray_ and the RR of mJOA score, with a higher R_gray_ associated with a lower mJOA score RR, but no correlation between IOUSI R_gray_ and preoperative mJOA score. Since IOUSI reflects the real-time status of the spinal cord after decompression instead of that of preoperative, this might explain why the IOUSI R_gray_ and preoperative mJOA score were not correlated. Nowadays, scientific community is focused toward the correlation of intraoperative findings with other preoperative biomarkers [35]. Although IOUS are affected by “interobserver variability” to be reliably used as a standalone biomarker, this intraoperative tool should be considered for the formulation, definition and validation of more sophisticated biosignatures. According to our experience about the application of IOUS, the IOUS can not only help us to evaluate and guide the decompression during the operation, but the gray value of IOUSI can be used to predict surgical outcomes, which may increase the application value of IOUS in spine surgery greatly. Based on the practicability of IOUS, we expect that the application of IOUS could be spread to more spinal surgical procedures. And we also hope the IOUSI R_gray_ could become a novel predictive indictor of surgical outcome for DCM in the future.

### Limitations

This study has the following limitations: as an exploratory study, the sample size was small; the follow-up period was relatively short; only the correlation between IOUSI and mJOA score were analyzed; and no assessments of patient-based outcomes was performed. Based on these preliminary results, long-term follow-up studies with a large sample should be carried out in the future. To obtain more accurate results, future studies should also include patient-based outcomes such as quality of life and quantitative performance tests such as the 10-s grip-and-release test and the 10-s step test.

## Conclusions

In DCM patients, the gray values of IOUSI can be measured accurately. The IOUSI R_gray_ correlated with postoperative neurological recovery significantly. Despite some limitations, to our best knowledge, this is the first mention of the concept of gray value of IOUSI in DCM and this prospective study was the first clinical study of the correlation between the gray values of IOUSI and neurological function in DCM patients. And we hope the IOUSI R_gray_ could become a novel predictive indictor of surgical outcome for DCM in the future.

## Data Availability

The datasets used and/or analysed during the current study are available from the corresponding author on reasonable request.

## Abbreviations

APD: Anteroposterior diameter
CSF: Cerebrospinal fluid
CR: Compression ratio
DCM: Degenerative cervical myelopathy
FDL: French-door laminoplasty
ISI: Increased signal intensity
IOUS: Intraoperative ultrasound
IOUSI: Intraoperative ultrasound imaging
MRI: Magnetic resonance imaging
MSCC: Maximum spinal cord compression
mJOA: Modified Japanese Orthopaedic Association
RR: Recovery rate
SCR: Signal change rate
TD: Traverse diameter
